# High-Sensitivity Cardiac Troponin T Is a Risk Factor for Major Adverse Cardiovascular Events and All-Cause Mortality: A 9.5-Year Follow-Up Study

**DOI:** 10.1155/2021/6647987

**Published:** 2021-08-25

**Authors:** Xiaona Wang, Peiqi Wang, Ruihua Cao, Xu Yang, Wenkai Xiao, Yun Zhang, Li Sheng, Ping Ye

**Affiliations:** ^1^Department of Geriatric Cardiology, The Second Medical Center and National Clinical Research Center for Geriatric Diseases, Chinese PLA General Hospital, Beijing, China; ^2^Department of Anesthesiology, The First Medical Center, Chinese PLA General Hospital, Beijing, China

## Abstract

**Background:**

The relationship between high-sensitivity cardiac troponin T (hs-cTnT) and different cardiovascular events has been observed in several large community studies, and the results have been controversial. However, there is currently no cross-sectional or longitudinal follow-up study on hs-cTnT in the Chinese population.

**Methods:**

We analyzed the association of plasma hs-cTnT levels with major adverse cardiovascular events (MACEs) and all-cause mortality in 1325 subjects from a longitudinal follow-up community-based population in Beijing, China.

**Results:**

In the Cox proportional hazards models analysis, the risk of MACEs increased with the increase of hs-cTnT levels (HR, 1.223, 95% CI, 1.054–1.418, *P*=0.008). Increased hs-cTnT levels were associated with coronary events (HR, 1.391, 95% CI, 1.106–1.749, *P*=0.005) in Model 4. Cox proportional risk regression model analysis revealed that increased hs-cTnT levels were associated with an increased risk of mortality (HR, 1.763, 95% CI, 1.224–2.540, *P*=0.002), even after adjusting hs-CRP and NT-proBNP. The area under the ROC curve for predicting MACEs was 0.559 (95% CI, 0.523–0.595, *P*=0.001). The areas under the ROC curve for predicting coronary events and mortality were 0.629 (95% CI, 0.580–0.678, *P* < 0.001) and 0.644 (95% CI, 0.564–0.725, *P* < 0.001), respectively.

**Conclusions:**

Our findings in the Chinese cohort support that hs-cTnT is a risk factor for major adverse cardiovascular events and all-cause mortality.

## 1. Introduction

It is extremely challenging to predict cardiovascular events in the general population; while the general population is unlikely to become a target of preventive measures, such measures have become a mainstay of how society addresses cardiovascular diseases [1]. Traditional risk factors such as hypertension, diabetes, hyperlipidemia, smoking, and obesity play important roles in the occurrence and development of cardiovascular disease. They can also be used as important prognostic factors for risk stratification and the prognostic evaluation of patients, but these traditional risk factors cannot be used to explain all prognostic risks. In recent years, new biomarkers, such as high-sensitivity cardiac troponin T (hs-cTnT) and nitrogen -terminated B-type brain key precursors (NT-proBNP), have been widely used in clinical practice and have been proven to be risk factors for cardiovascular diseases independent of traditional risk factors.

cTnT can be effectively used to discover subclinical heart disease and assess the risk of future cardiovascular disease, but the low detection rate of standard measurement methods limits its clinical application [2]. It is possible to measure cTnT with high sensitivity with the advancement of science and technology, and high-sensitivity cardiac troponin T (hs-cTnT) has emerged. Studies have found that the concentration of hs-cTnT in patients with stable coronary heart disease is significantly related to cardiovascular death or congestive heart failure [3, 4]. Subsequently, the prognostic value of hs-cTnT in the general population was also confirmed. A study involving 4,221 elderly community residents with a median follow-up time of 11.8 years found that baseline hs-cTnT and the change of hs-cTnT were significantly related to the incidence of heart failure and cardiovascular death [5]. Since then, the relationship between hs-cTnT and different cardiovascular events has been observed in several large community studies, and the results have been controversial [5–7]. New research has also shown differences in hs-cTnT levels among different ethnic groups. However, there is currently no cross-sectional or longitudinal follow-up study on hs-cTnT in the Chinese population.

Therefore, the current study examined the relationship of hs-cTnT with mortality and cardiovascular events by investigating a community population without the definite cardiovascular disease to ascertain the following: (1) the predictive relationship between the hs-cTnT level and mortality and cardiovascular events and (2) the predictive relationship between the change in hs-cTnT level and mortality and cardiovascular events in a large community-based longitudinal sample from China.

## 2. Methods

### 2.1. Subjects

After a routine health check-up between September 2007 and January 2009, a total of 1,680 subjects were initially eligible for cross-sectional analysis. During the follow-up from February 2013 to September 2013, 181 people were lost to follow-up due to various reasons. Finally, 1,499 subjects with complete data were included in the 5-year follow-up analysis (follow-up rate 89.2%). During the follow-up from June 2017 to September 2018, 174 people were lost to follow-up, and eventually, 1,325 subjects completed the follow-up (follow-up rate 89.2%). The research protocol of this project was approved by the ethics committee of the General Hospital of the PLA, and each subject provided informed written consent. The median follow-up interval for the original subjects was 9.5 years. During these visits, all participants received a questionnaire survey. Physical examinations, biochemical indicators, and biomarkers (including hs-cTnT) were reviewed at the same time. Any event reported was subject to verification through medical records, death certificates, pathological autopsy results, and objective coronary angiographic examination results and were jointly judged by two clinically experienced doctors.

### 2.2. Clinical Data Collection

Height (cm) and weight (kg) were measured. Systolic and diastolic blood pressures (SBP and DBP) were measured in the right arm twice in a sitting position after 5 min of rest. Blood samples were collected from participants after an overnight fast. Concentrations of fasting blood glucose (FBG), total cholesterol (TC), triglycerides (TGs), low-density lipoprotein cholesterol (LDL-C), high-density lipoprotein cholesterol (HDL-C), homocysteine (Hcy), and uric acid (UA) were detected on Roche Diagnostics GmbH (Roche Diagnostics GmbH, Mannheim, Germany). Serum creatinine (CR) was determined by a Roche enzyme assay kit on the Hitachi 7600 automatic analyzer (Hitachi, Tokyo, Japan). High-sensitivity C-reactive protein (hs-CRP) was detected on a Diension RxL Max analyzer (Siemens Healthcare Diagnostic) using an immunoassay method kit (Siemens Healthcare Diagnostic, USA, IN). N-Terminal B-type brain natriuretic peptide (NT-proBNP) was detected on an autoanalyzer using a Roche Diagnostics GmbH (Roche Diagnostics GmbH). High-sensitivity cardiac troponin T (hs-cTnT) was measured using the high-sensitivity Troponin T Elecsys kit (Roche Diagnostics GmbH, Mannheim, Germany). The concentration unit of hs-cTnT is pg/mL, and the coefficient of variation between batches is 8% at 10 pg/mL and 2.5% at 100 pg/mL [3].

### 2.3. Definition of Variables

Body mass index (BMI): weight (kg)/height^2^ (m^2^).

Hypertension: SBP ≥140 mmHg and/or DBP 90 mmHg or those who have taken antihypertensive drugs.

Diabetes mellitus: fasting venous blood glucose ≥7.0 mmol/L, oral glucose tolerance test (OGTT) with 2 h blood glucose ≥11.1 mmol/L, symptoms of hyperglycemia and random blood glucose ≥11.1 mmol/L, or receiving hypoglycemic treatment [8].

Estimated glomerular filtration rate (eGFR): 141 × min (Scr/k,1) *α* × max (Scr/k,1)−1.209 × 0.993 age × 1.018 (if female)  × 1.159 (if black), where Scr is plasma creatinine (mg/dL), *k* is 0.7 for females and 0.9 for males, *α* is −0.329 for females and −0.411 for males, min indicates the minimum of Scr/k or 1, and max indicates the maximum of Scr/k or 1.

hs-cTnT: the lowest detectable concentration according to the kit instructions is 3 pg/mL (according to the reagent instructions), which is used as the cut-off value in this study. Therefore, hs-cTNT levels ≥3 pg/mL are considered measurable. The 99th percentile for the hs-cTnT of a group of healthy people aged 20 to 70 years was 14 pg/mL [9], and hs-cTnT concentrations ≥14 pg/mL were generally considered to be elevated.

All-cause mortality was determined by a review of death certificates. The definition of MACEs comprised nonfatal myocardial infarction, newly diagnosed CHD (identified by coronary artery imaging or receiving coronary revascularization), stroke (ischemic or hemorrhagic), and cardiovascular mortality.

Cardiovascular death was defined as deaths related to atherosclerotic heart disease (fatal myocardial infarction and definite fatal coronary heart disease), cerebrovascular disease deaths (fatal stroke), and causes including heart failure death from other atherosclerotic and cardiovascular diseases [10].

MACEs were defined as cardiovascular death, nonfatal myocardial infarction, coronary revascularization, coronary heart disease, and stroke confirmed by coronary imaging [11].

Coronary heart disease (CHD) events were defined as coronary heart disease death, nonfatal myocardial infarction, coronary revascularization, and coronary heart disease diagnosed by coronary imaging.

Stroke was defined as acute, focal damage to the central nervous system caused by vascular causes, which results in neurological deficits, including cerebral infarction (rather than internal hemorrhage) and subarachnoid hemorrhage [12].

### 2.4. Statistical Analyses

Continuous variables are expressed as the mean or median (interquartile range) ± standard deviation (SD) and dichotomous variables are expressed as percentages. Analysis of continuous variables was performed by the *t*-test, and analysis of categorical variables was performed by *χ*^2^ test.

Pearson regression analysis and stepwise multivariate linear regression analysis were performed to evaluate the associations between baseline hs-cTnT and baseline traditional cardiovascular risk factors.

For the analyses of hs-cTnT as a categorical variable, subjects were divided into three groups according to their baseline hs-cTnT level: hs-cTnT <3 pg/mL, hs-cTnT between 3 and 14 pg/mL, and hs-cTnT ≥14 pg/mL. The relationship between baseline hs-cTnT levels and MACEs and coronary events and mortality was analyzed by Cox proportional hazards models. Models were defined as follows: model 1 = adjusted for age and gender; model 2 = adjusted for model 1 + presence of hypertension or diabetes mellitus, current smoking status, SBP, postprandial blood glucose, TC, HDL-C, antihypertensive medication use, and antidiabetic medication use; model 3 = adjusted for model 2 + eGFR; model 4 = adjusted for model 3 + hs-CRP and NT-proBNP (both after logarithmic transformation).

We also analyzed the relationship between hs-cTnT as a continuous variable and endpoints, in which values of cTnT that were below the detection limit were assigned to 1.5 ng/L (i.e., one-half of the lower limit of detection).

SPSS17.0 software was used for all statistical analyses. *P* value < 0.05 was considered statistically significant.

## 3. Results

### 3.1. General Characteristics of the Study Population

The general characteristics of the population are shown in Table 1. In this population, the average age was 59.10 ± 9.8 years, and 51.5% were females. Of these 1,325 people, 736 had a detectable level of hs-cTnT (>3.0 pg/ml), accounting for 55.54% of the total, as shown in Table 1. The distribution range of hs-cTnT was from 3.03 pg/ml to 176.4 pg/ml, and the median was 7.36 pg/ml (25% and 75% digits were 4.76 pg/ml and 15.58 pg/ml, respectively). There were 143 people with an increased hs-cTnT (≥14.0 pg/ml), accounting for 10.79% of the total. Comparing the three groups, the cardiovascular risk factors in the middle and increased groups were significantly higher than those in the lower group. In addition, cardiovascular risk factors, such as being male, history of diabetes, history of hypertension, FBG, uric acid, Hcy, in the increased group were significantly different from those in the other two groups.

### 3.2. Clinical Factors Affecting Hs-cTnT

At baseline, univariate analysis showed that elderly (*r* = 0.085, *P*=0.004), male (*r* = 0.120, *P* < 0.001), smoking (*r* = 0.110, *P*=0.001), hypertension (*r* = 0.278, *P* < 0.001), Scr (*r* = 0.101, *P*=0.001), FBG (*r* = 0.065, *P*=0.026), BNP (*r* = 0.119, *P* < 0.001), and hs-CRP (*r* = 0.087, *P*=0.005) were positively correlated with hs-cTnT; eGFR (*r* = −0.091, *P*=0.002) was negatively correlated with hs-cTnT. Multivariate linear regression analysis showed that male (*β* = 0.725, *P* < 0.001), hypertension (*β* = 0.883, *P* < 0.001), FBG (*β* = 0.064, *P* < 0.001), TC (*β* = 0.227, *P* < 0.001), and LDL-C (*β* = 0.222, *P*=0.003) were positively correlated with hs-cTnT, while eGFR (*β* = −0.596, *P* < 0.001) was negatively correlated with hs-cTnT (Table 2).

### 3.3. Associations of Baseline Hs-cTnT Levels with Major Adverse Cardiovascular Events (MACEs), Coronary Events, and Mortality

**Figure 1:**
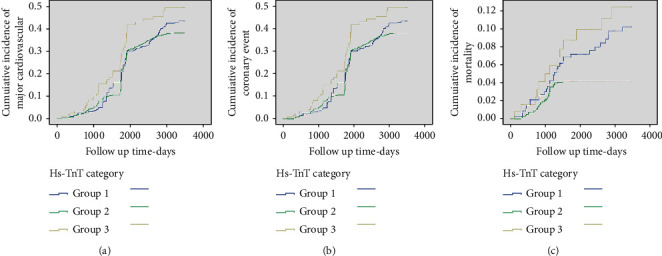
Risk for cardiovascular events and all-cause mortality by baseline hs-cTnT level. Kaplan–Meier survival curves indicating cumulative incidence of major adverse cardiovascular events (a), coronary event (b), and all-cause mortality (c) across baseline hs-cTnT categories.

During the follow-up period, a total of 191 participants experienced MACEs, and the incidence increased from 11.05% in the lowest (hs-cTnT < 3 pg/ml) group to 23.02% in the highest group (hs-cTnT > 14 pg/ml), as demonstrated by Kaplan–Meier survival analysis (*P* < 0.001) (Figure 1(a)). In addition, after adjusting for multiple factors in the Cox proportional hazards models analysis (Model 4), the risk of MACEs increased with the increase of hs-cTnT levels (HR, 1.223, 95% CI, 1.054–1.418, *P*=0.008) (Table 3).

Similar trends were discovered for coronary events. A total of 121 participants experienced coronary events, and the risk for coronary events and all-cause mortality by baseline hs-cTnT level is shown in Figure 1(b). Increased hs-cTnT levels were associated with coronary events (HR, 1.391, 95% CI, 1.106–1.749, *P*=0.005) in Model 4 (Table 3).

During the median follow-up of 9.5 years, a total of 84 deaths occurred, and the mortality rate increased significantly from 8.08% in the lowest quartile group (hs-cTnT <3 pg/mL) to 10.32% in the highest quartile (hs-cTnT >14 pg/mL) (*P* < 0.01) (Figure 1(c)). After adjusting for age, gender, blood pressure, blood lipids, renal function, and other traditional cardiovascular risk factors, Cox proportional risk regression model analysis revealed that increased hs-cTnT levels were associated with an increased risk of mortality (HR, 1.763, 95% CI, 1.224–2.540, *P*=0.002), even after adjusting hs-CRP and NT-proBNP (Model 4) (Table 3).

### 3.4. Hs-cTnT Predicts MACEs and Mortality

**Figure 2:**
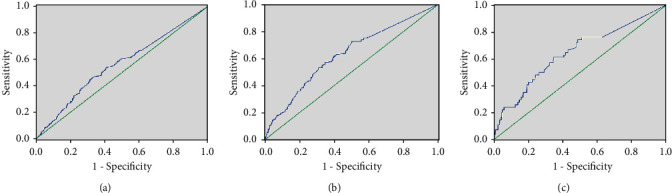
Hs-TnT predicts the ROC curve of major adverse cardiovascular events (a), coronary event (b), and all-cause mortality (c). The area under the curve (AUC) is (a) 0.559 (95% confidence interval: 0.523–0.595, *P*=0.001); (b) 0.629 (95% confidence interval: 0.580–0.678, *P* < 0.001); and (c) 0.644 (95% confidence interval: 0.564–0.725, *P* < 0.001), respectively.

The ROC curve was used to analyze the accuracy of hs-cTnT in predicting MACEs (Figures 2(a)–2(c)). The area under the ROC curve for predicting MACEs was 0.559 (95% CI, 0.523–0.595, *P*=0.001) (Figure 2(a)). The best cut-off value of hs-cTnT for predicting MACEs was 5.01 pg/mL. At the same time, the sensitivity and specificity were 53.8% and 59.1%, respectively.

The areas under the ROC curve for predicting coronary events and mortality were 0.629 (95% CI, 0.580–0.678, *P* < 0.001) (Figure 2(b)) and 0.644 (95% CI, 0.564–0.725, *P* < 0.001) (Figure 2(c)), respectively. The best cut-off value of hs-cTnT for predicting coronary events and mortality were 4.05 pg/mL and 4.6 pg/mL, respectively. When the hs-cTnT value was 4.05 pg/mL, the sensitivity for predicting coronary events was 72.5%, and the specificity was 49.8%. When the hs-cTnT value was 4.6 pg/mL, the sensitivity for predicting mortality was 74.1%, and the specificity was 51.4%.

## 4. Discussion

To our knowledge, this study is the first follow-up study to evaluate the predictive value of hs-cTnT for cardiovascular events and mortality conducted in China. Through the follow-up study of this community population for nearly 10 years, this study has some significance. First, we observed minor subclinical myocardial injury in the general population using the latest generation of hs-cTnT measurement methods, rather than only focusing on the elderly or high-risk population. We found that the hs-cTnT detection rate in this population was 54.70%. Second, the results confirmed that the baseline level of hs-cTnT can predict the mortality and cardiovascular event risk of the population of the community. Furthermore, we also found that increases in hs-cTnT also have predictive value for cardiovascular events and mortality.

In recent years, highly sensitive methods for detecting hs-cTnT have increased in clinical practice. At present, there is not a very clear definition of hs-cTnT, which is mainly based on the analytical performance of the lowest detection limit and the measurement precision in the low cTnT concentration range. The definition of hs-cTnT is as follows [13–15]: high-sensitivity methods can detect cTnT (e.g., as low as 10 ng/L) levels that cannot be detected by current traditional methods; the cTnT with the minimum detection value of CV ≤ 10% and the 99th percentile value detected by the system or reagent that meets the requirements of the guidelines; or the cTnT can be detected in some or all of the surface healthy people and the 99th percentile value/CV ≤ 10%. In 2018, the International Federation of Clinical Chemistry and Laboratory Medicine released the latest specific quality standards for troponin detection [16], which proposed that high-sensitivity troponin should meet the detection rate of more than 50% in apparently healthy males and females. New research has also shown differences in hs-cTnT levels among different ethnic groups [17, 18]. The results of this study suggest that the prevalence of hs-cTnT levels >14.0 pg/mL was approximately 11.0%, which is slightly higher than the previous studies. In the current study, the detection rate of hs-cTnT in the general population in the Beijing community was 70.65% for males and 43.85% for females, respectively, which was similar to foreign studies. Our findings are slightly different from the previous study, probably because of the middle-aged and elderly in the study. Although there is no clear history of cardiovascular disease, traditional cardiovascular risk factors, such as hypertension, diabetes, and smoking, are still common in this group of middle-aged and elderly people. Undiagnosed resting myocardial ischemia and chronic changes in cardiac structure and function may still possibly be present.

hs-cTnT has been recognized as the first marker of myocardial injury in the diagnosis, risk stratification, and prognosis of an acute coronary syndrome (ACS), and its value has been recognized at home and abroad [19]. Gareth et al. concluded that early elevation in plasma hs-cTnT within 24 h of elective noncardiac surgery precedes the subsequent development of noncardiac organ dysfunction [20]. Because of its high sensitivity and specificity, hs-cTnT has been agreed upon by the European Heart Association and Chinese experts as the best basis for early diagnosis of acute myocardial infarction (AMI) and risk stratification of heart diseases [21]. In predicting future events, multiple trials have confirmed an association between elevated hs-cTnT levels and mortality or cardiovascular death in some populations at high risk for cardiovascular disease. In addition, few studies have reported the association of hs-cTnT levels with cardiovascular events in individuals from a general population, and the results have been controversial. The Atherosclerosis Risk in Communities (ARIC) Study found an association between detectable cTnT and a highly sensitive assay was associated with incident CHD, mortality, and HF in individuals without known CHD/stroke [22]. Evidence from a prospective study of the cTnT detected with a highly sensitive assay supports a stronger association with structural heart disease and subsequent risk for all-cause mortality [6]. Changes in cTnT levels measured with a highly sensitive assay were found to be significantly associated with incident HF and cardiovascular death [7]. Welsh did not identify a significant relationship between baseline cTnT levels and some CVD outcomes, whereas they reported that cTnT is more strongly associated with the risk of non-CVD death [23]. Although hs-cTnT as a continuous variable is significantly related to MACEs and mortality, when used as a categorical variable, the clear correlation between hs-cTnT and mortality was only present in the highest group (>14 pg/mL). In the Cox proportional hazard model analysis, the correlation between hs-cTnT and MACEs and mortality did not decrease significantly after adjusting for traditional risk factors, renal function, and hs-CRP. However, when the NT-proBNP level was further adjusted, the risk of MACEs and mortality was significantly reduced, suggesting that NT-proBNP and hs-cTnT partially overlap information about abnormalities in cardiac structure and function.

Few studies have reported the optimal hs-cTnT cut-off for predicting MACEs and mortality in the general population. The use of an equivalent cut-off for hs-cTnT merits further discussion, and universally using a cut-off of 0.014 *μ*g/L may result in the improper diagnosis of acute CVD [24]. An individual patient data meta-analysis reported that the optimal cut-off value for all-cause death was 18 ng/L in a chronic heart failure population, whereas the optimal cut-off values increased progressively with worse renal function [25]. In our study, the ROC curve analysis predicted the area under the ROC curve for MACEs and mortality was 0.559 and 0.644, respectively. When the hs-cTnT value was 5.01 pg/mL, the sensitivity for predicting MACEs was 53.8% and the specificity was 59.1%. When the hs-cTnT value was 4.6 pg/mL, the sensitivity for predicting MACEs was 74.1% and the specificity was 51.4%. Obviously, specialized research should determine whether patient management guided by the same cut-off value is beneficial to prognosis and cost-effective.

The mechanisms of small elevations of hs-cTnT in apparently healthy subjects are not fully understood. It is well known that serum elevation of cTnT is associated with myocardial ischemia, and a positive correlation between elevated hs-cTnT levels and new or suspected ECG ST-T changes or new left bundle branch block was recently described [4]. However, the study found that concentrations of hs-cTnT remained unchanged after exercise in patients with and without detectable ischemia [26], supporting that factors (e.g., coronary microvascular dysfunction [27], apoptosis [28, 29], or subclinical abnormalities of cardiac structure or function [30] could induce troponin release) in addition to ischemia contribute significantly to the risk associated with cTnT. Therefore, there may be several mechanisms underlying elevated troponin levels, and it is unclear which mechanisms are related to the outcomes observed in our study.

A new study shows that incorporating hs-cTnI testing into risk algorithms for patients with arteriosclerotic cardiovascular disease (ASCVD) provides enhanced risk stratification and leads to the reclassification of about 12% of patients into a more appropriate risk group [31]. Troponin levels should not be viewed just as a marker for myocardial injury and diagnosis of MI in acute coronary syndrome but should be used more frequently for assessing CVD risk in stable patients with ischemic heart disease. It is very meaningful to screen hs-cTnT in the general population. Elevated hs-cTnT levels in community populations may be an early warning device to reflect the risk of chronic cardiovascular disease or future cardiovascular disease. However, the increase of sensitivity may also lead to the decrease of diagnostic specificity, especially in diabetes, chronic kidney disease, or elderly men, where an increase of the hs-cTnT level is very common. Therefore, it is necessary to establish appropriate hs-cTnT diagnostic thresholds according to different populations. The deficiency of this study is the lack of objective examination to evaluate the structure and function of the heart, such as echocardiography and imaging examinations of coronary arteries.

## 5. Conclusions

Our findings in the Chinese cohort support that hs-cTnT is a risk factor for major adverse cardiovascular events and all-cause mortality.

## Figures and Tables

**Table 1:** Baseline characteristics and laboratory test results of subjects.

	Hs-TnT group (ng/L)			
	Group 1 (*n* = 589)	Group 2 (*n* = 593)	Group 3 (*n* = 143)	*P* value
Age (year)	58.4 ± 12.3	60.7 ± 11.7	62.7 ± 11.1	0.010
Male (*n* (%))	155 (26.31%)	308 (51.94%)	89 (62.2%)	0.001
BMI (kg/m^2^)	25.4 ± 3.8	25.5 ± 3.6	25.6 ± 3.7	0.872
Smoking (n (%))	51 (8.66%)	108 (18.21%)	30(20.97%)	0.022
SBP (mmHg)	131.8 ± 15.6	132.7 ± 17.4	134.7 ± 16.5	0.893
DBP (mmHg)	77.2 ± 12.6	77.1 ± 11.7	77.6 ± 12.5	0.275
TC (mmol/L)	5.05 ± 0.91	5.03 ± 0.93	4.89 ± 0.92	0.259
TG (mmol/L)	1.92 ± 1.69	1.76 ± 1.09	2.07 ± 1.83	0.018
HDL-C (mmol/L)	1.34 ± 0.45	1.38 ± 0.36	1.33 ± 0.32	0.298
LDL-C (mmol/L)	2.96 ± 0.75	2.92 ± 0.71	2.86 ± 0.74	0.512
FBG (mmol/L)	5.63 ± 2.24	5.29 ± 1.46	5.85 ± 2.22	<0.001
Scr (mmol/L)	67.61 ± 15.91	66.27 ± 16.48	73.49 ± 20.29	<0.001
eGFR (ml/min^−1^/1.73 m^2^)	90.57 ± 14.83	87.38 ± 13.73	85.14 ± 13.51	<0.001
hs-CRP (mg/L)	2.4 (1.3, 3.4)	2.3 (1.4, 3.4)	2.4 (1.6, 3.6)	0.365
BNP	35.51 ± 15.03	38.32 ± 10.95	48.55 ± 18.37	<0.001
Hs-TnT (pg/mL)		5.24 ± 2.79	26.06 ± 17.94	<0.001

TC, total cholesterol; HDL-C, high-density lipoprotein cholesterol; TG, triglyceride; LDL-C, low-density lipoprotein cholesterol; SBP, systolic blood pressure; DBP, diastolic blood pressure; BMI, body mass index; FBG, fast blood glucose; eGFR, estimated glomerular filtration rate; HR, heart rate; Scr, serum creatinine; hs-CRP, high-sensitive C-reactive protein; BNP, brain natriuretic peptide; Hs-TnT, high-sensitivity troponin T.

**Table 2:** Clinical factors affecting Hs-TnT.

All subjects (*n* = 1325)	Hs-TnT			Hs-TnT	
	*r*	*P* value	*β*	CI	*P* value
Age	0.085	0.004	0.002	−0.002∼0.007	0.308
Male	0.120	<0.001	0.725	0.340∼1.110	<0.001
Smoking	0.110	0.001	0.320	−0.086∼0.725	0.122
Diabetes	0.144	<0.001	0.232	0.058∼0.522	0.117
Hypertension	0.278	<0.001	0.883	0.501∼1.265	<0.001
TG^*∗*^	0.013	0.646	0.044	−0.002∼0.091	0.063
HDL-C^*∗*^	0.029	0.321	0.100	−0.070∼0.270	0.250
LDL-C	0.019	0.515	0.222	0.077∼0.367	0.003
TC	0.049	0.092	0.227	0.102∼0.351	<0.001
SBP	0.011	0.702	0.046	0.033∼0.060	0.872
DBP	0.049	0.093	0.002	0.000∼0.003	0.010
BMI	0.033	0.258	0.010	−0.002∼0.007	0.003
FBG	0.065	0.026	0.064	0.037∼0.091	<0.001
Cr	0.101	0.001	0.231	0.098∼0.391	0.565
eGFR^*∗*^	−0.091	0.002	-0.596	−0.915∼−0.277	<0.001
hs-CRP	0.087	0.005	0.044	−0.009∼0.097	0.105
BNP	0.119	<0.001	0.016	−0.022∼0.055	0.411

TC, total cholesterol; HDL-C, high-density lipoprotein cholesterol; TG, triglyceride; LDL-C, low-density lipoprotein cholesterol; SBP, systolic blood pressure; DBP, diastolic blood pressure; BMI, body mass index; FBG, fast blood glucose; eGFR, estimated glomerular filtration rate; HR, heart rate; Scr, serum creatinine; hs-CRP, high-sensitive C-reactive protein; BNP, brain natriuretic peptide; Hs-TnT, high-sensitivity troponin T. ^*∗*^Natural logarithm transformed. §Covariates in the multiple-adjusted models included age, gender, hypertension, diabetes, current smoking, and levels of plasma.

**Table 3:** Cox proportional hazards models analysis for associations between baseline hs-cTnT levels and outcomes.

	Mace		Coronary event		All-cause mortality	
	HR (95% CI)	*P* value	HR (95% CI)	*P* value	HR (95% CI)	*P* value
Unjust	1.313 (1.144–1.506)	<0.001	1.747 (1.440–2.210)	<0.001	1.996 (1.470–2.709)	<0.001
Model1	1.188 (1.026–1.375)	0.021	1.395 (1.122–1.734)	0.003	1.472 (1.025–2.115)	0.036
Model2	1.183 (1.016–1.377)	0.030	1.392 (1.110–1.746)	0.004	1.657 (1.145–2.397)	0.007
Model3	1.223 (1.054–1.418)	0.008	1.391 (1.106–1.749)	0.005	1.763 (1.224–2.540)	0.002

Models are defined as follows: model 1 = adjusted for age and gender; model 2 = adjusted for model 1 + presence of hypertension or diabetes mellitus, current smoking status, systolic blood pressure, postprandial blood glucose, total cholesterol, high-density lipoprotein cholesterol, antihypertensive medication use, and antidiabetic medication use; model 3 = adjusted for model 2 + estimated glomerular filtration rate; model 4 = adjusted for model 3 + high-sensitivity C-reactive protein and N-terminal pro-B-type natriuretic peptide (both after logarithmic transformation). MACE, major adverse cardiovascular event.

## Data Availability

The data used to support the study are available from the corresponding author upon request.

## Abbreviations

hs-cTnT: High-sensitivity cardiac troponin T
SBP: Systolic blood pressures
DBP: Diastolic blood pressures
FBG: Fasting blood glucose
TC: Total cholesterol
TGs: Triglycerides
LDL-C: Low-density lipoprotein cholesterol
HDL-C: High-density lipoprotein cholesterol
Hcy: Homocysteine
hs-CRP: High-sensitivity C-reactive protein
NT-proBNP: N-Terminal B-type brain natriuretic peptide
MACEs: Major adverse cardiovascular events
ASCVD: Arteriosclerotic cardiovascular disease.
